# A predictive model based on routine blood inflammatory markers for IgA vasculitis in children

**DOI:** 10.3389/fped.2026.1843776

**Published:** 2026-07-28

**Authors:** Li Cao, Xin Huang, Zhi-Huan Chen, Xiao-Qiang Lian

**Affiliations:** 1 Department of Clinical Laboratory, Handan Center Hospital, Handan, China; 2 Handan Center for Disease Control and Prevention, Handan, China; 3 School of Special Education, Handan University, Handan, China

**Keywords:** IgA vasculitis, lymphocyte, neutrophil, NLR, PLR

## Abstract

**Background:**

IgA vasculitis (IgAV) is the most prevalent systemic vasculitis in children, however, there is still a lack of sensitive biomarkers for its diagnosis. This study aims to analyse the association between blood count-derived inflammatory markers and the onset and progression of pediatric IgAV, thereby providing novel reference criteria for early disease identification and risk warning.

**Methods:**

Pediatric patients diagnosed with IgAV at Handan Central Hospital between June 2022 and September 2023 formed the case group, matched with healthy children as the control group. Blood counts were analysed using the Sysmex XN3000 fully automated haematology analyser. Logistic regression screened risk factors and constructed a diagnostic nomogram model, with model efficacy assessed via ROC curves, calibration curves, and decision curve analysis (DCA).

**Results:**

Significant differences were observed between the two groups in neutrophil count, platelet count, neutrophil percentage, lymphocyte percentage, neutrophil-to-lymphocyte ratio (NLR), and platelet-to-lymphocyte ratio (PLR). Multivariate regression analysis showed that the combination of NLR and PLR was an independent risk factor for childhood IgAV. A nomogram integrated with age, gender, NLR, and PLR was further constructed. Evaluation via calibration curves and clinical decision curve analysis (DCA) demonstrated that this predictive model has some potential as an auxiliary diagnostic tool for the early detection of the disease.

**Conclusions:**

The results of this study indicate that NLR and PLR are significantly associated with the onset of IgAV in children. The combined nomogram model based on these two indicators serves only as a preliminary exploratory tool for a single center. It relies solely on routine complete blood counts for quantitative assessment and can assist clinicians in conducting early, preliminary screening for IgAV.

## 1 Introduction

IgA vasculitis (IgAV), formerly termed Henoch–Schönlein purpura (HSP), is a type of leukocytoclastic vasculitis affecting small vessels, characterised by neutrophilic infiltration (1). It represents the most common systemic vasculitis in children, with approximately 90% of cases occurring in children aged 5 to 10 years (2). The pathogenesis is complex, closely associated with infection, genetic predisposition, and environmental factors. The core pathological mechanism involves abnormal IgA antibody production triggered by mucosal immune dysregulation and the deposition of immune complexes within the vascular wall, potentially affecting multiple organs including the skin, gastrointestinal tract, joints, and kidneys. Current clinical management of IgA vasculitis involves stratification based on clinical presentation and organ involvement. Mild cases may be managed with rest and symptomatic supportive care, whereas severe organ involvement necessitates combination therapy with glucocorticoids, immunosuppressants, or even targeted biologics. Concurrently, infection prophylaxis and long-term monitoring of renal function and urine routine are critical measures for improving prognosis (3, 4).

Assessing disease activity and predicting adverse outcomes early are key priorities in the clinical management of IgA vasculitis. In recent years, alongside classical clinical and pathological diagnosis, the exploration of efficient, readily accessible biomarkers has become a research focus. The neutrophil-to-lymphocyte ratio (NLR) and platelet-to-lymphocyte ratio (PLR), as systemic inflammatory markers derived from routine blood counts, have garnered widespread attention due to their ease of testing and low cost. Their prognostic value has been established in numerous adult inflammatory and neoplastic diseases, and this concept is gradually extending to the pediatric field (5–8). Current research confirms their association with IgAV disease activity, identifying them as potential independent risk factors for renal involvement and prognosis in pediatric IgAV (9). However, the predictive efficacy of single inflammatory markers remains limited, failing to comprehensively reflect the complex pathophysiological processes of the disease. Consequently, constructing precise early-risk prediction models using multiple combined indicators has become a core focus and pressing challenge in current IgAV clinical research. Accordingly, this study aims to establish an early risk prediction model for pediatric IgAV centred on NLR and PLR, validating its predictive reliability and clinical applicability. This endeavour seeks to identify more representative inflammatory biomarkers, thereby providing objective and disease activity assessment, early risk stratification, and personalised follow-up management.

## 2 Materials and methods

### 2.1 Study population

This study was a retrospective case-control investigation. The case cohort comprised pediatric patients with IgAV who presented for initial consultation at the Department of pediatrics, Handan Central Hospital from June 2022 to September 2023. Inclusion criteria: Meeting the 2010 IgAV diagnostic criteria established by the European League Against Rheumatism/Pediatric Rheumatology International Trial Organisation/European pediatric Rheumatology Society (10); Age below 12 years; No prior use of immunosuppressants or corticosteroids; 4Presence of prominent purpura on limbs or buttocks. Exclusion criteria: Immunodeficiency disorders; Haematological malignancies; Concurrent cardiac, hepatic, or renal organ damage; Associated leukopenia or thrombocytopenia; Incomplete clinical records. Children undergoing routine health examinations at the same hospital during the same period served as the control group, matched 1:1 with the case group. Ultimately, 101 cases and 101 controls were enrolled, comprising 202 subjects in total.

### 2.2 Data collection

Data were collected on age, gender, onset time, and haematological parameters at initial hospital examination for children in both the case group and the healthy control group. Haematological parameters included white blood cell count (WBC), neutrophil percentage, lymphocyte percentage, monocyte percentage, absolute neutrophil count, absolute lymphocyte count, absolute monocyte count, red blood cell count (RBC), haematocrit (HCT), haemoglobin (HGB), platelet count (PLT), and mean platelet volume (MPV). The neutrophil-to-lymphocyte ratio (NLR) and platelet-to-lymphocyte ratio (PLR) were calculated from laboratory data. complete blood count (CBC) data were obtained using the Sysmex XN3000 automated haematology analyser.

### 2.3 Statistical analysis

Data analysis was performed using R software version 4.2.1. Quantitative data are expressed as mean ± standard deviation (±s) and categorical data as frequency (*n*) and percentage (%). Comparisons between groups for quantitative data were conducted using t-tests, while categorical data comparisons employed *χ*^2^ tests. To identify independent risk factors for pediatric IgAV, baseline data were first compared between groups. Multivariate logistic regression analysis was then employed to determine independent predictors. Based on these selected risk factors, a risk prediction nomogram model for pediatric IgAV was constructed. The topmost section of this nomogram displays the individual scores for each variable, enabling separate scoring for each variable. The bottom section shows the total score, which is the sum of all individual variable scores. The total score for a study subject can be compared against the disease probabilit*y* axis at the bottom of the nomogram to determine their probability of developing IgAV. It is worth noting that within the risk chart, variables with a broader score distribution exert greater predictive influence on the model. The NLR + PLR combination exhibits the widest scoring range, making it the most influential predictor in the model.

The diagnostic efficacy of this nomogram model and its individual predictive indicators was assessed using receiver operating characteristic (ROC) curves. Calibration curves were plotted to evaluate the nomogram model's calibration by comparing the consistency between the model's predicted probabilities and the actual observed clinical probabilities at different quantiles. The decision curve analysis (DCA) plot was generated using the “rmda” software package. The clinical utility of this column nomogram model was assessed by calculating the model's net clinical benefit (NCB).

Following study completion, *post-hoc* power analysis was conducted using G*Power 3.1 software. Results indicated a test power of 0.99 at *α* = 0.05 for primary statistical analyses, confirming adequate sample size and robust statistical power. *P* < 0.05 was considered statistically significant.

## 3 Results

### 3.1 Baseline characteristics

There were 202 subjects enrolled in this study, comprising 101 in the healthy group and 101 in the case group. Baseline characteristic analysis revealed that the neutrophil count, platelet count, neutrophil percentage (Neu%), NLR, and PLR were significantly higher in the case group than in the control group, whereas the lymphocyte percentage (Lym%) was significantly lower. All differences were statistically significant (*P* < 0.05). Detailed data are shown in Table 1.

**Table 1:** Comparison of general characteristics between the two groups of patients.

name	Healthy group (*N* = 101)	Case group (*N* = 101)	Total (*N* = 202)	*P*
Age	7.48 ± 2.77	7.96 ± 2.40	7.72 ± 2.60	0.185
Gender				
Male	53 (52.5%)	63 (62.4%)	116 (57.4%)	0.200
Female	48 (47.5%)	38 (37.6%)	86 (42.6%)	
Neutrophil count	3.25 ± 1.55	5.24 ± 3.90	4.25 ± 3.12	<0.001
Lymphocyte count	3.03 ± 1.21	2.80 ± 1.06	2.92 ± 1.14	0.153
Monocyte count	0.44 ± 0.20	0.53 ± 0.40	0.48 ± 0.32	0.049
Platelets	268.71 ± 74.84	325.96 ± 93.68	297.34 ± 89.31	<0.001
Mean platelet volume	9.13 ± 1.03	9.25 ± 1.44	9.19 ± 1.25	0.477
Neutrophil %	46.05 ± 12.45	56.40 ± 13.83	51.23 ± 14.11	<0.001
Lymphocyte%	44.35 ± 12.26	35.59 ± 13.27	39.97 ± 13.48	<0.001
Monocyte%	6.42 ± 2.16	6.12 ± 1.80	6.27 ± 1.99	0.292
NLR	1.28 ± 1.04	2.14 ± 1.80	1.71 ± 1.53	<0.001
PLR	106.26 ± 68.15	128.95 ± 53.84	117.61 ± 62.31	0.009

NLR, neutrophil/lymphocyte ratio; PLR, platelet/lymphocyte ratio.

### 3.2 Logistic regression analysis of risk factors for IgAV

**Figure 1:**
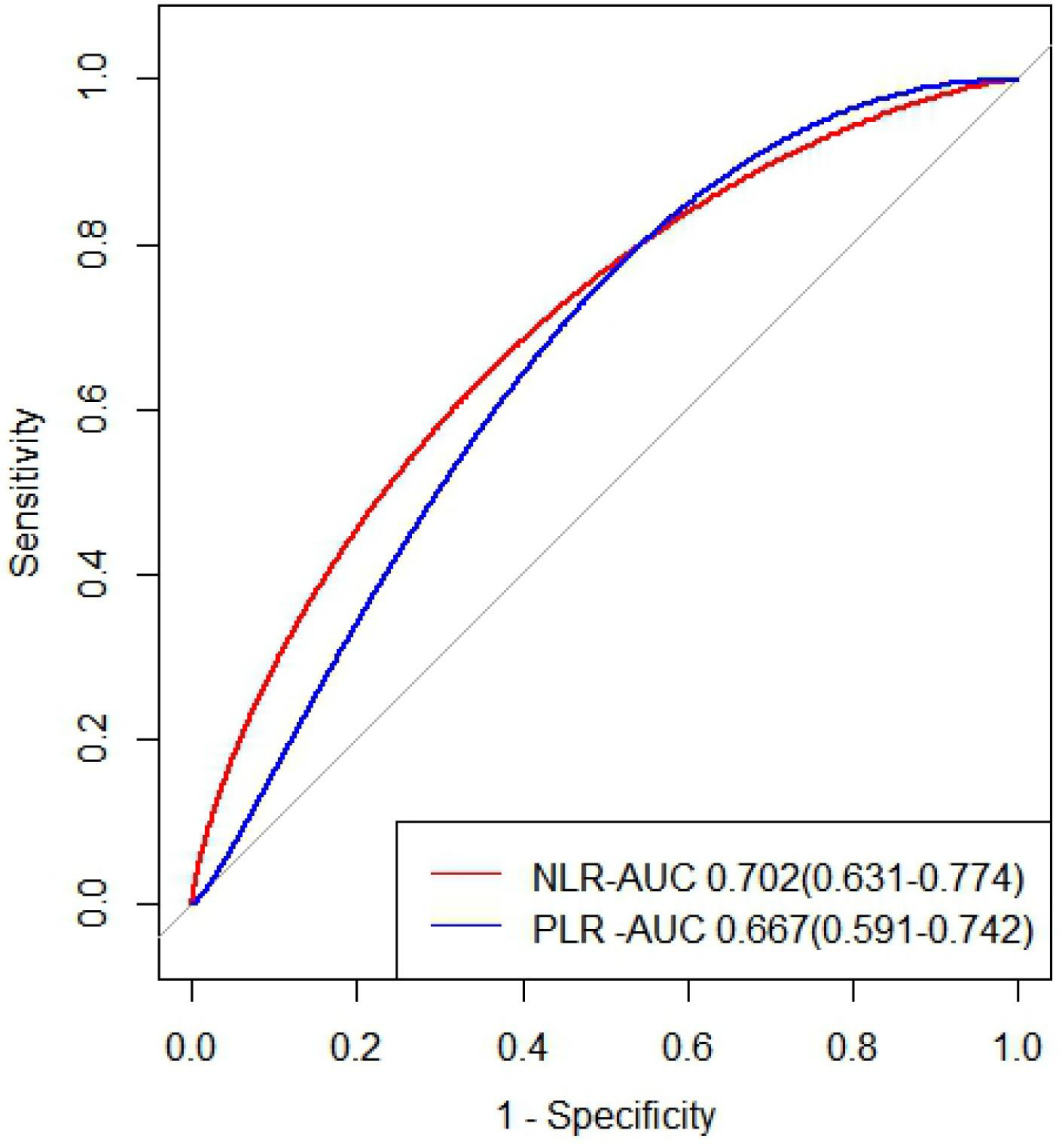
Diagnostic efficacy of NLR and PLR in IgAV.

**Figure 2:**
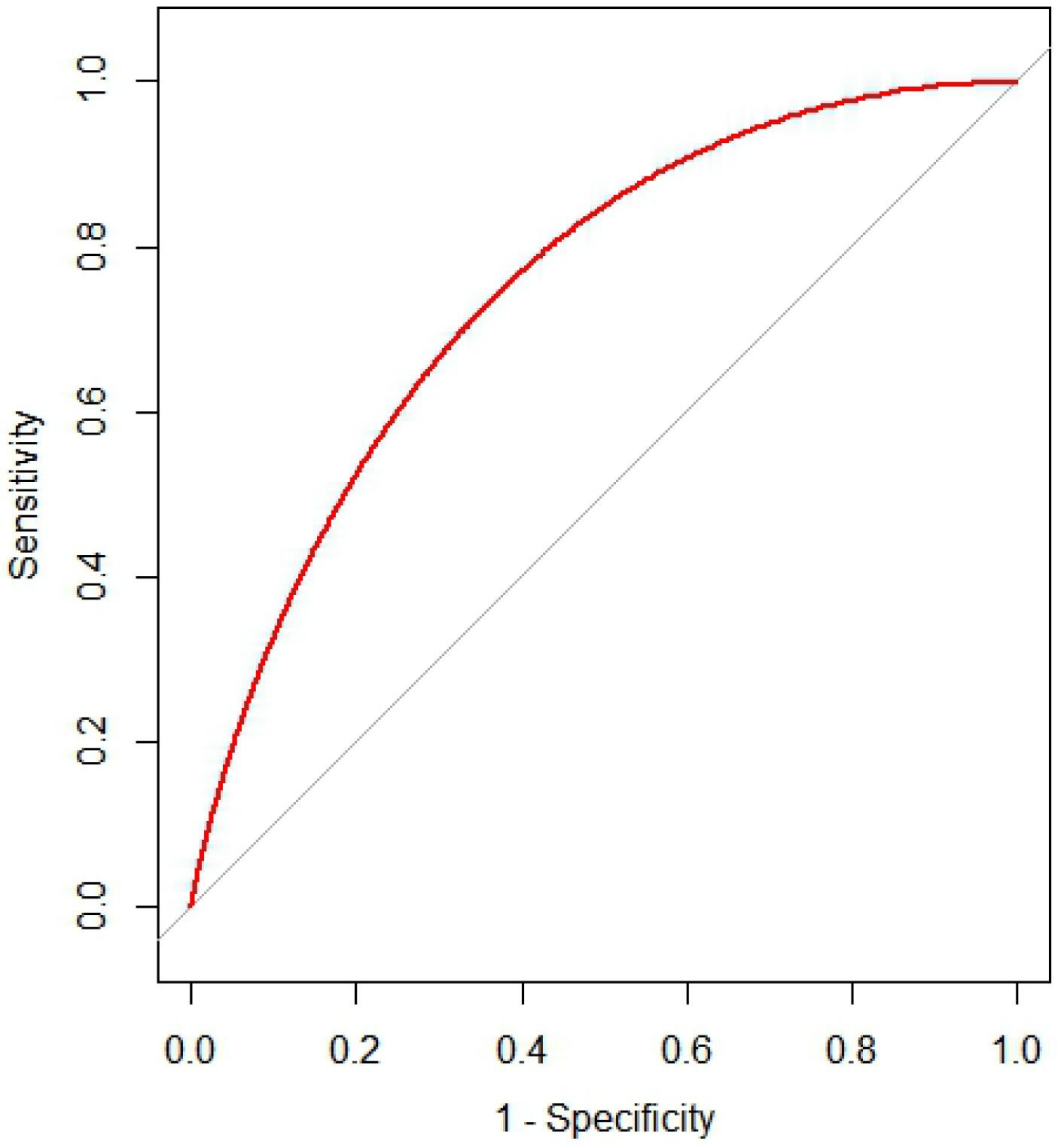
ROC curve analysis for the nomogram model.

The results of the multivariate logistic regression analysis revealed that Neu%, Lym%, NLR and PLR were all independent risk factors for the development of IgAV in children (Table 2). ROC curve analysis revealed that the AUC for the NLR was 0.702 (95% CI: 0.631–0.774, *P* < 0.001), and the AUC for the PLR was 0.667 (95% CI: 0.591–0.742, *P* < 0.001) (Figure 1). NLR: Optimal cut-off: 3.26 (Maximum Youden index 0.335, Sensitivity 68.4%, Specificity 65.1%). PLR: Optimal cut-off: 142.5 (Maximum Youden index 0.246, Sensitivity 63.2%, Specificity 61.4%). Further analysis of the predictive efficacy of the combined index (NLR + PLR) revealed an AUC of 0.756 (95% CI: 0.682–0.830, *P* < 0.001), corresponding to a specificity of 71.3% and a sensitivity of 73.3%. This diagnostic performance significantly surpassed that of either individual marker (Figure 2).

**Table 2:** Logistic regression analysis of risk factors for HSP.

Characteristic	Regression coefficient (*β*)	Standard Error	OR1	95% CI1	*p*-value
Age	0.077	0.061	1.08	0.97, 1.20	0.2
Gender (ref: male)					
Female	−0.4	0.326	0.67	0.38, 1.16	0.2
Neutrophil %	0.058	0.012	1.06	1.04, 1.09	<0.001
Lymphocyte %	−0.051	0.013	0.95	0.92, 0.97	<0.001
NLR	0.56	0.155	1.75	1.33, 2.41	<0.001
PLR	0.01	0.004	1.01	1.00, 1.01	0.013
NLR + PLR grading (ref: 0)					
Grade 1	0.944	0.412	2.57	1.08, 6.47	0.037
Grade 2	2.118	0.443	8.31	3.81, 19.60	<0.001

NLR, neutrophil/lymphocyte ratio; PLR, platelet/lymphocyte ratio.

### 3.3 Development of a risk prediction nomogram model for IgAV

**Figure 3:**
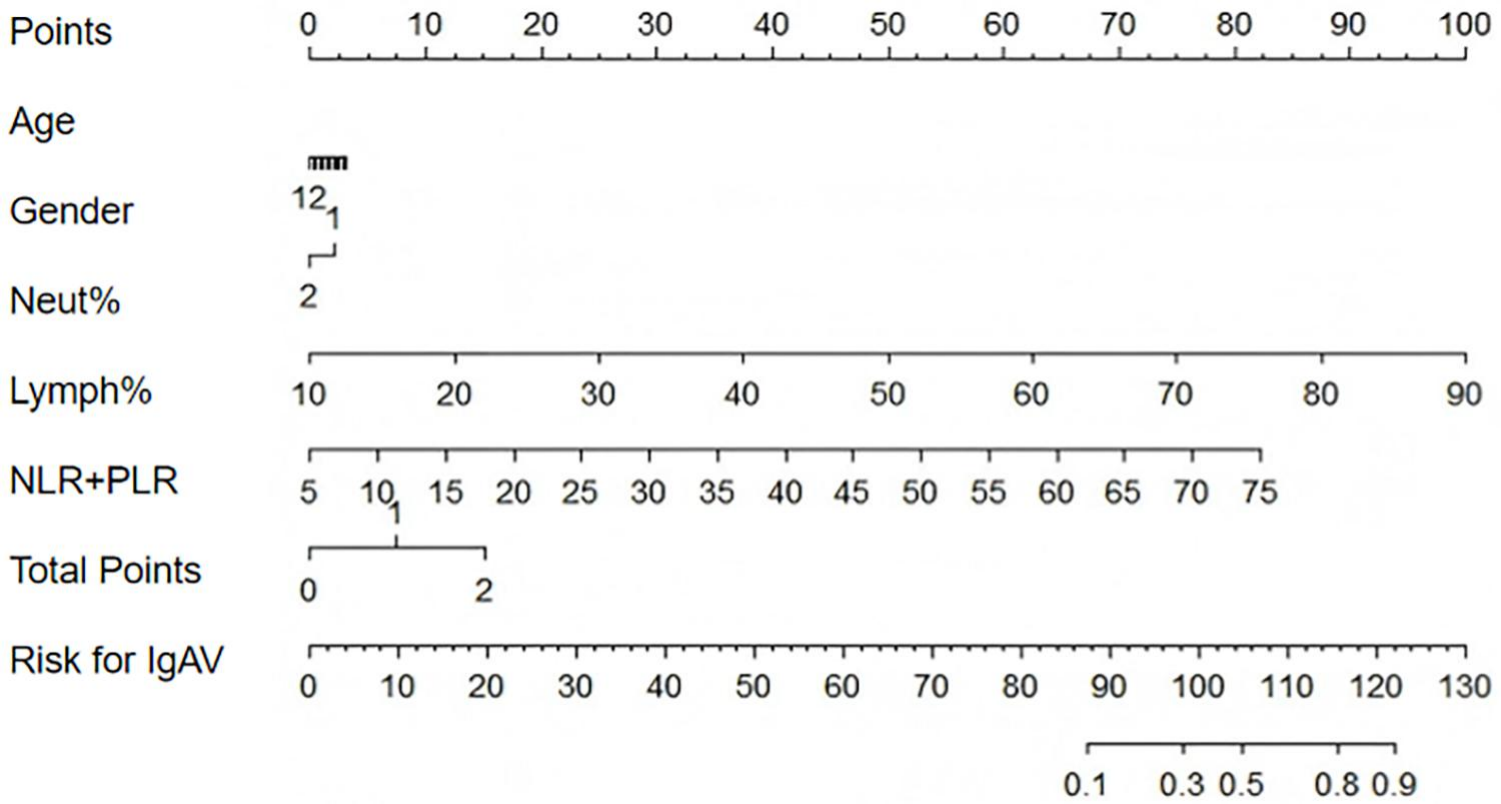
Risk prediction nomogram model for IgAV.

Based on the results of multifactorial logistic regression analysis, a risk stratification model incorporating five independent predictors—age, sex, Neu%, Lym%, and the combined NLR + PLR index—was constructed for childhood IgA vasculitis (Figure 3). To quantify the risk contribution of the combined NLR + PLR index, this study established the following scoring rules: a score of 2 was assigned if both NLR and PLR exceeded their respective cut-off thresholds; a score of 1 was assigned when only one indicator was elevated; and a score of 0 if neither indicator was elevated. Details are presented in Table 2.

The left side of the Nomogram chart displays the varying assigned values for the five predictor variables, the centre shows the corresponding integral axis, and the right side presents the probabilit*y* axis for IgAV occurrence risk (0%–100%). Each predictor variable in the model is assigned an independent integral scoring rule, meaning every actual clinical value of the variable corresponds to a unique integral value within the column chart. The variable's influence on IgAV occurrence risk is positively correlated with its integral score -a higher assigned score indicates a greater contribution of that variable level to the risk of IgAV occurrence. Among these, the NLR + PLR combination possesses the broadest score assignment range, making it the most influential predictor in this model.

### 3.4 Validation and clinical utility assessment of the IgAV nomogram model

**Figure 4:**
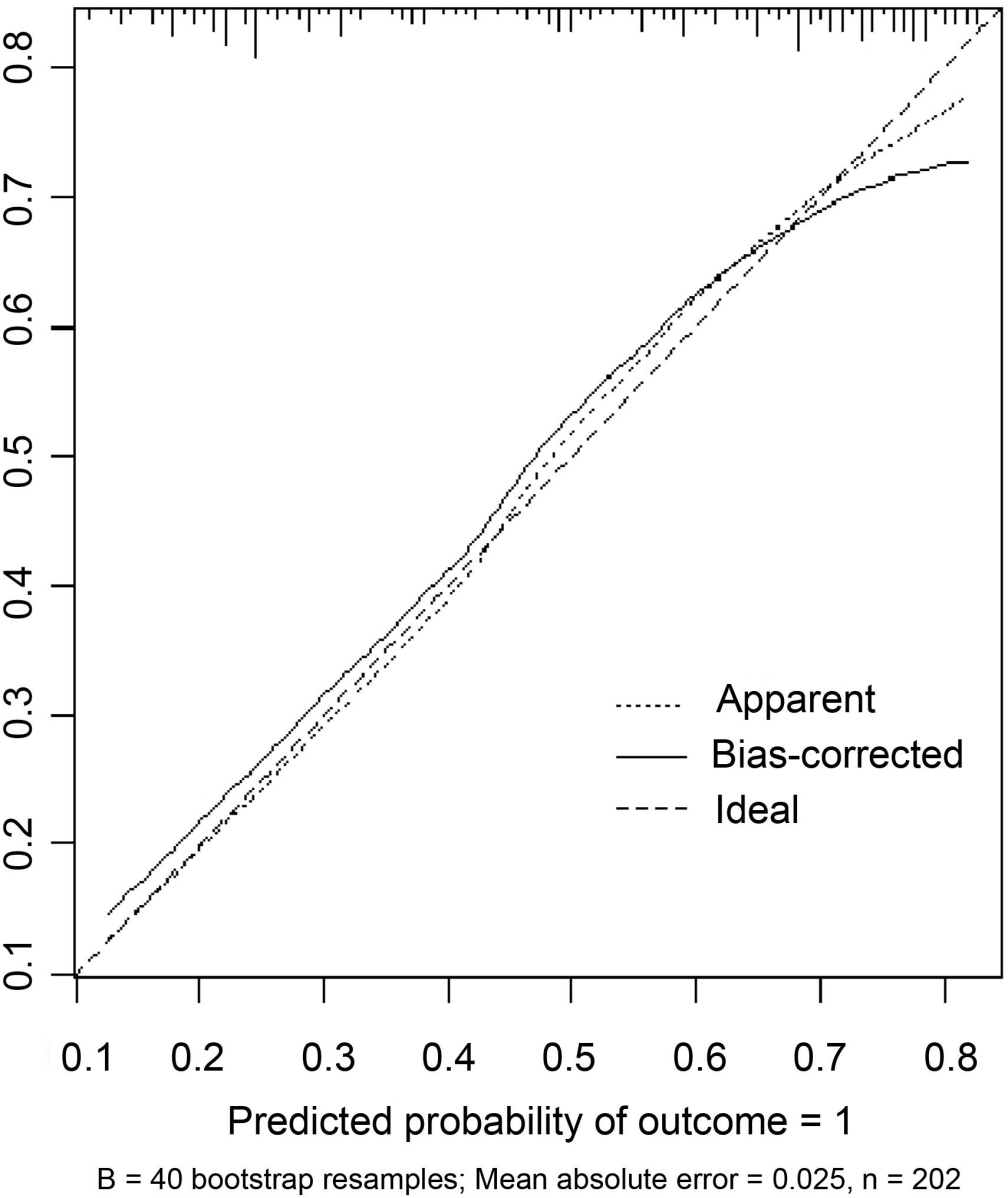
Model calibration curve.

Internal validation of the risk nomogram model was conducted with Bootstrap resampling performed 40 times. Results showed that the calibration curve closely approximated the ideal curve (diagonal line), indicating high concordance between predicted and actual probabilities (Figure 4). A calibration slope of 0.042 and an intercept of 0.961. A slope close to 1 and an intercept close to 0 indicate that the model's predicted probabilities align well with the actual incidence rates; the Brier score of 0.118 suggests that prediction errors are small and that the model's calibration is optimal.

**Figure 5:**
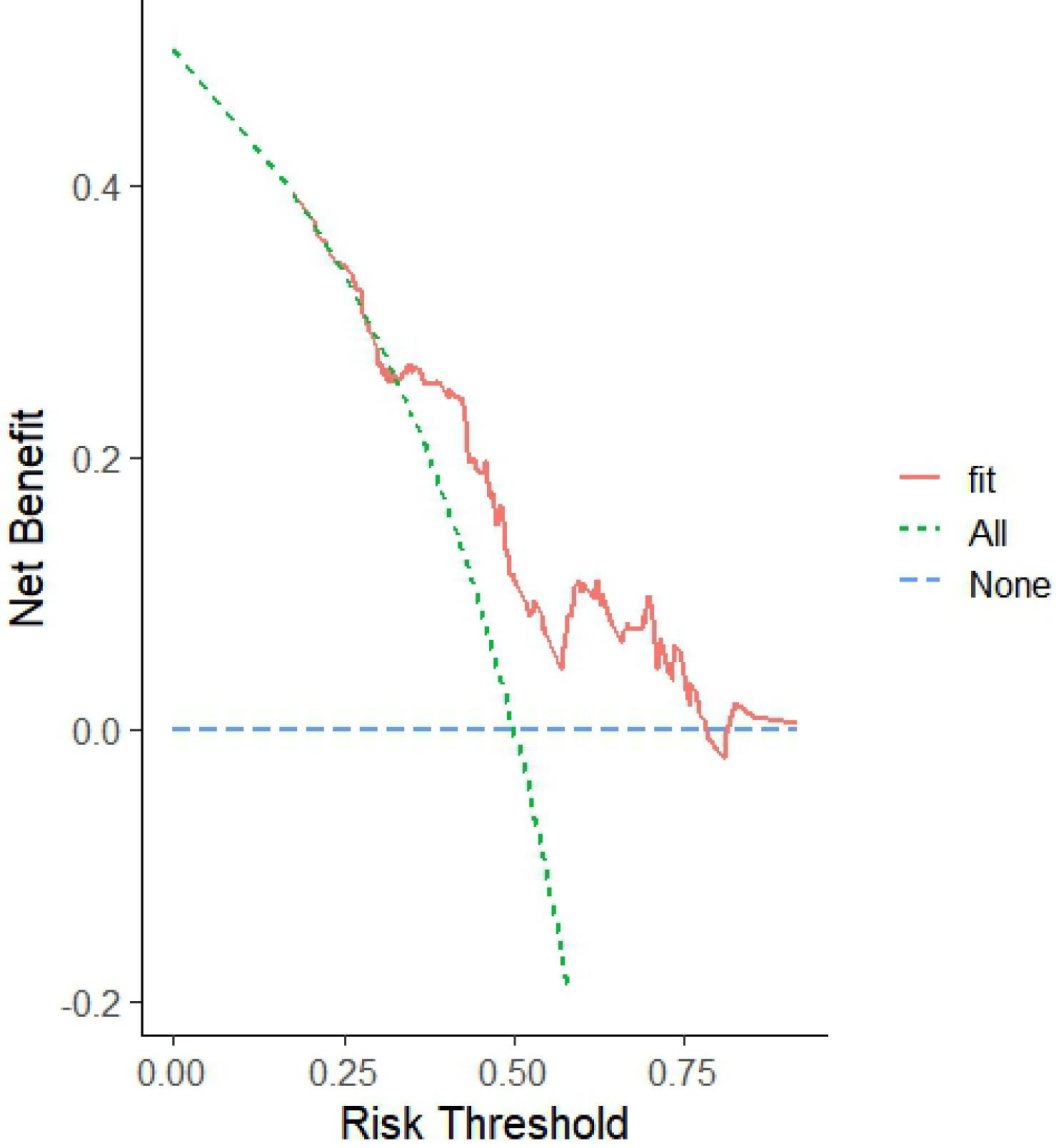
DCA curve analysis of the nomogram.

The occurrence of IgAV in patients was selected as the state variable, with the risk prediction value obtained from the nomogram serving as the test variable. The decision curve analysis (DCA) curve indicates that when the prediction probability threshold of the nomogram model ranges from 0.00 to 0.80, the net benefit lies between 0 and 0.5 (Figure 5). The net clinical benefit of the nomogram model consistently exceeded that of both the “full intervention” and “no intervention” strategies.

## 4 Discussion

Previous Studies have established a robust pathophysiological basis for the involvement of NLR and PLR in IgAV pathogenesis: the core mechanism of IgAV involves small vessel inflammation triggered by immune complex deposition, with infection serving as a primary precipitating factor that disrupts the balance between innate and adaptive immunity (11). Regarding NLR, prior studies confirm that its elevation reflects an imbalance between the innate pro-inflammatory response, represented by neutrophils, and lymphocyte-mediated adaptive immune regulation. This imbalance not only creates a favourable local microenvironment for IgA1 immune complex formation and deposition but also directly reflects systemic inflammatory activation levels (12, 13). Compared to acute-phase proteins such as C-reactive protein (CRP) and Interleukin-6 (IL-6), NLR more accurately reflects the core immune dysfunction in IgA vasculitis and correlates closely with disease activity (14). Regarding the PLR, platelets in IgAV perform dual roles in inflammation and haemostasis regulation: they function as effector cells in vascular inflammation while also serving as key executors in microthrombus formation and exacerbated vascular injury. By inducing a hypercoagulable state, they promote immune complex deposition and organ damage (15, 16). Previous studies indicate that an elevated PLR not only suggests platelet activation and lymphocyte apoptosis triggered by the release of inflammatory mediators following vascular injury (17, 18), but also directly reflects abnormal activation of the coagulation pathway, being highly correlated with the pathophysiology of severe complications such as renal involvement in IgAV (19). This study further confirms that both NLR and PLR levels in pediatric IgAV patients are significantly elevated compared to healthy controls. This highlights the distinct value of these ratios in reflecting disease pathophysiology from the perspectives of “immune imbalance” and “inflammation-coagulation interaction”, respectively, providing theoretical support for combined modelling.

NLR and PLR respectively reflect the pathophysiological characteristics of IgAV from the dimensions of immune imbalance and inflammation-coagulation interaction, with their complementary nature providing theoretical justification for combined modelling. The NLR-PLR combined predictive model developed in this study overcomes the limitations of single-marker efficacy. Validation demonstrates its robust early predictive value, confirming that combined markers more comprehensively capture IgAV's pathological sequence of “inflammatory activation-immune imbalance-coagulation abnormalities-vascular injury”. Compared to single markers, this approach better reflects the disease's overall activity state. Age and gender constitute fundamental demographic factors influencing the onset of pediatric IgAV, with their predictive value confirmed by multiple studies. The peak incidence of pediatric IgAV occurs during the pre-school and school-age periods (4–7 years). The developmental characteristics of the immune system during this age range result in a more active immune response to pathogen infections, predisposing children to vasculitic reactions. Regarding gender differences, male patients slightly outnumber females, and males are more likely to present with gastrointestinal and renal involvement. This may be related to differences in the level of immune development between boys and girls (20). Neu% and Lym% constitute core indicators in peripheral blood counts, with their variations directly reflecting the acute-phase immune inflammatory response state in pediatric patients. As an immune-mediated systemic small-vessel vasculitis, IgAV hinges on the activation of inflammatory cytokines. During the acute phase, patients typically exhibit elevated neutrophil percentages and relatively reduced lymphocyte percentages. This imbalance in the granulocyte-lymphocyte ratio sensitively reflects the body's inflammatory burden (21). The NLR + PLR combination represents a composite inflammatory marker derived from blood counts, gaining significant attention in IgAV research in recent years. NLRand PLR provide a more comprehensive reflection of overall systemic inflammation and platelet activation status (22). This study confirms that NLR and PLR values in IgAV pediatric patients during the acute phase are significantly higher than in healthy controls. Abnormal platelet activation constitutes a pivotal pathophysiological process in IgAV-related vascular injury. Activated platelets release multiple inflammatory mediators, promoting vascular endothelial damage and immune complex deposition (13, 14). Consequently, the combined NLR-PLR index—as a composite indicator integrating the neutrophil, lymphocyte, and platelet pathways—emerges as a core predictive factor in this model.

The Nomogram model in this paper employs a method whereby single-variable integrals are summed to yield a total integral, which is then used to determine the corresponding risk probability. The specific operational steps are as follows: ① Based on the clinical actual data of the pediatric research subject, locate the subject's indicator value on the corresponding variable axis on the left side of the column-line graph. Project vertically upwards to the central integral axis and read the single integral value corresponding to that variable; ② After sequentially completing the integration readings for all five variables, sum all individual integration values to obtain the total integration value; ③ Precisely locate the total integration value on the total integration axis at the top of the column-line graph, projecting vertically downwards to the IgAV occurrence probabilit*y* axis on the right. The numerical value at the projection point corresponds to the specific probability of IgAV occurrence for that research subject. The chart selection comprises clinical standardised routine blood count parameters, offering convenient testing with high reproducibility. This effectively mitigates variations in testing across different healthcare institutions. The model's intuitive operation and quantifiable results enable both individualised risk assessment for IgA vasculitis and enhanced suitability for primary care settings and routine clinical practice (23, 24). This substantially elevates the model's clinical utility, aligning with current clinical demand for convenient, efficient predictive tools. Given the case–control design with artificially set prevalence, the absolute net benefit from DCA cannot be directly applied to clinical decision-making. Nevertheless, the model consistently outperformed the “no intervention” strategy across different threshold probabilities, suggesting its potential value for preliminary screening. Prospective cohort studies are required in the future to verify whether this model-guided clinical pathway can improve patient outcomes.

Current research on inflammatory biomarkers in IgAV predominantly focuses on the diagnostic and prognostic value of individual markers. While some studies have attempted to construct predictive models, these primarily target complications such as gastrointestinal system involvement (22) and renal inpairment (25, 26). Furthermore, they frequently incorporate non-routine, high-cost testing indicators, limiting their applicability in early disease screening and risk stratification. For instance, Li et al.'s meta-analysis (12) reviewed retrospective case-control studies that concentrated solely on the diagnostic value of single ratio indicators, failing to explore the synergistic effects of multi-marker combinations. The advantage of this study lies in being the first to propose a nomogram model for the early diagnosis of IgAV in children based on blood routine-based inflammatory markers (NLR combined with PLR). Unlike the study by Jiang YJ et al. (27), which focused on predicting IgAV recurrence, this study shifts the research emphasis to early disease identification and warning. The model is entirely based on routine blood tests, offering advantages such as lower cost and faster acquisition, making it particularly suitable for rapid screening in outpatient or primary healthcare settings. For example, studies by SUN et al. (28) and Bi Y et al. (29) respectively developed predictive models for gastrointestinal haemorrhage in pediatric patients with abdominal HSP and for nephritis in IgA vasculitis patients. Although the predictive targets (complications) in these studies differ from the disease diagnosis in the present investigation, their approach of employing multi-factor modelling is analogous to that adopted here. However, these models incorporate numerous indicators, and certain parameters (such as APTT, IgE, D-dimer, complement C4, and IgG) are not routine or low-cost tests, thereby limiting their practicality for rapid clinical initial screening.

This study has inherent limitations. The retrospective case-control design may cause selection bias, and prospective validation covering IgAV patients with different severity levels is needed. We did not adjust for infection history, fever, antibiotic use, C-reactive protein (CRP) and erythrocyte sedimentation rate (ESR) (30), so elevated NLR and PLR may reflect general inflammation rather than IgAV-specific changes. The 1:1 matched design artificially balanced cases and controls, leading to an unrealistic prevalence. The predicted probabilities from the nomogram cannot be regarded as absolute population risks and should be interpreted cautiously in clinical practice. Moreover, single-center recruitment limited sample size and generalizability. With only healthy controls, the model can only screen high-risk populations and cannot distinguish IgAV from other febrile infectious diseases. Future multi-center prospective studies will include patients of all severities and disease control groups, incorporate more clinical indicators, and perform external validation to improve model performance.However, the strength of this study lies in its focus on the early risk prediction of IgAV disease in children. A combined model was constructed using low-cost, readily available parameters derived from routine blood tests as its core. This not only compensates for the limitations of single-parameter prediction but also better meets the practical needs of early clinical screening, thereby providing new insights for research into IgAV biomarkers.

In summary, NLR and PLR reflect the pathophysiological characteristics of paediatric IgAV from different perspectives. The early-risk prediction model constructed by combining these two parameters demonstrates a certain degree of discriminatory power and potential clinical applicability, providing a quantitative reference indicator for assessing disease activity and early risk stratification in paediatric IgAV. As this model is based on routine complete blood count results, it is relatively simple to implement and has a sound basis for wider adoption, thereby assisting clinicians in identifying paediatric patients at potential high risk. It provides supplementary information for personalised intervention and follow-up management, which may play a positive role in reducing the risk of severe organ involvement and improving long-term outcomes for paediatric patients. It is worth noting that this model represents a key advancement in predicting the renal risk of IgA vasculitis in children. However, as preliminary results from a single-center retrospective study, this model should be used only for preliminary research and exploration pending independent external validation, and is not yet suitable for direct implementation in large-scale routine clinical practice.

## Data Availability

The raw data supporting the conclusions of this article will be made available by the authors, without undue reservation.

## References

[1] Jennette JC Falk RJ Bacon PA Basu N Cid MC Ferrario F. 2012 revised international Chapel Hill consensus conference nomenclature of vasculitides. Arthritis Rheum. (2013) 65(1):1–11. 10.1002/art.37715 23045170

[2] Reicy R Jari M. Comparison of different treatment regimens for long-term improvement of renal function in patients with Henoch-Schönlein Purpura: a systematic review. Curr Rheumatol Rev. (2024) 20(1):57–64. 10.2174/1573397119666230825163008 37698064

[3] Dawod ST Akl KF. Henoch-Schöenlein syndrome in Qatar: the effects of steroid therapy and paucity of renal involvement. Ann Trop Paediatr. (1990) 10(3):279–84. 10.1080/02724936.1990.11747443 1703745

[4] Oni L Sampath S. Childhood IgA vasculitis (Henoch Schonlein Purpura)-advances and knowledge gaps. Front Pediatr. (2019) 7:257. 10.3389/fped.2019.00257 31316952 PMC6610473

[5] Cho J Liang S Lim SHH Lateef A Tay SH Mak A. Neutrophil to lymphocyte ratio and platelet to lymphocyte ratio reflect disease activity and flares in patients with systemic lupus erythematosus-A prospective study. Jt Bone Spine. (2022) 89(4):105342. 10.1016/j.jbspin.2022.105342 35032639

[6] Ochi H Kurosaki M Joko K Mashiba T Tamaki N Tsuchiya K. Usefulness of neutrophil-to-lymphocyte ratio in predicting progression and survival outcomes after atezolizumab-bevacizumab treatment for hepatocellular carcinoma. Hepatol Res. (2023) 53(1):61–71. 10.1111/hepr.13836 36070216

[7] Hahn S Kim K-M Kim M-J Choi H-S Noh H Cho I-J. Usefulness of hounsfield units and the Serum neutrophil-to-lymphocyte ratio as prognostic factors in patients with breast cancer. Cancers (Basel). (2022) 14(14):3322. 10.3390/cancers14143322 35884383 PMC9318691

[8] Yuan W Tian Y Lin C Wang Y Liu Z Zhao Y. Pectic polysaccharides derived from Hainan Rauwolfia ameliorate NLR family pyrin domain-containing 3-mediated colonic epithelial cell pyroptosis in ulcerative colitis. Physiol Genomics. (2023) 55(1):27–40. 10.1152/physiolgenomics.00081.2022 36440907

[9] Gayret OB Erol M Tekin Nacaroglu H. The relationship of neutrophil-lymphocyte ratio and platelet-lymphocyte ratio with gastrointestinal bleeding in Henoch-Schonlein Purpura. Iran J Pediatr. (2016) 26(5):e8191. 10.5812/ijp.8191 28203340 PMC5297442

[10] Ozen S Pistorio A Iusan SM Bakkaloglu A Herlin T Brik R. EULAR/PRINTO/PRES criteria for Henoch-Schönlein purpura, childhood polyarteritis nodosa, childhood Wegener granulomatosis and childhood Takayasu arteritis: Ankara 2008. Part II: final classification criteria. Ann Rheum Dis. (2010) 69(5):798–806. 10.1136/ard.2009.116657 20413568

[11] Liu C Luo L Fu M Li Z Liu J. Analysis of children with Henoch-Schonlein purpura secondary to infection. Clin Rheumatol. (2022) 41(3):803–10. 10.1007/s10067-021-06007-9 34993728

[12] Li B Ren Q Ling J Tao Z Yang X Li Y. Clinical relevance of neutrophil-to-lymphocyte ratio and mean platelet volume in pediatric Henoch-Schonlein Purpura: a meta-analysis. Bioengineered. (2021) 12(1):286–95. 10.1080/21655979.2020.1865607 33412982 PMC8291875

[13] Lei W Yun-Yun S Ai-E X. Neutrophil-to-lymphocyte ratio: a biomarker for predicting systemic involvement in Henoch-Schonlein Purpura. Indian J Dermatol Venereol Leprol. (2021) 88(1):132. 10.25259/IJDVL_760_19 34623056

[14] Özdemir ZC Çetin N Kar YD Öcal HO Bilgin M Bör Ö. Hemotologic indices for predicting internal organ involvement in Henoch-Schönlein Purpura (IgA vasculitis). J Pediatr Hematol Oncol. (2020) 42(1):e46–9. 10.1097/MPH.0000000000001571 31851146

[15] Chang D Cheng Y Luo R Zhang C Zuo M Xu Y. The prognostic value of platelet-to-lymphocyte ratio on the long-term renal survival in patients with IgA nephropathy. Int Urol Nephrol. (2021) 53(3):523–30. 10.1007/s11255-020-02651-3 33113085 PMC7906929

[16] Shao WX Ye Q Wang XJ. Application value of laboratory indexes in the differential diagnosis of Henoch-Schoenlein purpura. Praktischer Wert von Laborindizes bei der differenzialdiagnose der Purpura Schoenlein-Henoch. Z Rheumatol. (2017) 76(4):351–6. 10.1007/s00393-016-0108-0 27444626

[17] Cai J Wang K Han T Jiang H. Evaluation of prognostic values of inflammation-based makers in patients with HBV-related acute-on-chronic liver failure. Medicine (Baltimore). (2018) 97(46):e13324. 10.1097/MD.0000000000013324 30431619 PMC6257482

[18] Karadağ ŞG Çakmak F Çil B Tanatar A Sönmez HE Kıyak A. The relevance of practical laboratory markers in predicting gastrointestinal and renal involvement in children with Henoch-Schönlein Purpura. Postgrad Med. (2021) 133(3):272–7. 10.1080/00325481.2020.1807161 32772751

[19] Toraman A Neşe N Özyurt BC Kürşat S. Association between neutrophil-lymphocyte & platelet lymphocyte ratios with prognosis & mortality in rapidly progressive glomerulonephritis. Indian J Med Res. (2019) 150(4):399–406. 10.4103/ijmr.IJMR_1234_17 31823922 PMC6902366

[20] Atchariyaphuk N Sukharomana M Chaiyapak T Charuvanij S. Recurrent immunoglobulin A vasculitis in children and adolescents: prevalence and associated risk factors. Clin Exp Pediatr. (2026) 69(1):46–55. 10.3345/cep.2025.01158 41125223 PMC12790900

[21] Jaszczura M Góra A Grzywna-Rozenek E Barć-Czarnecka M Machura E. Analysis of neutrophil to lymphocyte ratio, platelet to lymphocyte ratio and mean platelet volume to platelet count ratio in children with acute stage of immunoglobulin A vasculitis and assessment of their suitability for predicting the course of the disease. Rheumatol Int. (2019) 39(5):869–78. 10.1007/s00296-019-04274-z 30868223

[22] Guo Q Xia S Ding Y Liu F. Predictive laboratory markers for gastrointestinal complications in children with Henoch-Schönlein Purpura. J Multidiscip Healthc. (2025) 18:279–88. 10.2147/JMDH.S499808 39866350 PMC11761534

[23] Shi J Ye Y Zhu D Su L Huang Y Huang J. Automatic segmentation of cardiac magnetic resonance images based on multi-input fusion network. Comput Methods Programs Biomed. (2021) 209:106323. 10.1016/j.cmpb.2021.106323 34365312

[24] Schwalm JD Di S Sheth T Natarajan MK O’Brien E McCready T. A machine learning-based clinical decision support algorithm for reducing unnecessary coronary angiograms. Cardiovasc Digit Health J. (2021) 3(1):21–30. 10.1016/j.cvdhj.2021.12.001 35265932 PMC8890355

[25] Sugino H Sawada Y Nakamura M. Iga vasculitis: etiology, treatment, biomarkers and epigenetic changes. Int J Mol Sci. (2021) 22(14):7538. 10.3390/ijms22147538 34299162 PMC8307949

[26] Gong Y-Q Han L Zhang J-Y Yu J Wu N Hu W-P. Abdominal imaging and endoscopic characteristics of adult abdominal IgA vasculitis: a multicenter retrospective study. Ann Med. (2024) 56(1):2408467. 10.1080/07853890.2024.2408467 39324401 PMC11429444

[27] Jiang YJ Song DY Li JL. Predictive value of complete blood count and inflammation marker on risk of recurrence in children with Henoch-Schönlein Purpura. Zhongguo Shi Yan Xue Ye Xue Za Zhi. (2023) 31(3):837–42. 10.19746/j.cnki.issn.1009-2137.2023.03.032 37356948

[28] Sun L Liu W Li C Zhang Y Shi Y. Construction and internal validation of a predictive model for risk of gastrointestinal bleeding in children with abdominal Henoch-Schönlein purpura: a single-center retrospective case-control study. Front Immunol. (2022) 13:1025335. 10.3389/fimmu.2022.1025335 36248897 PMC9556720

[29] Bi Y Quan W Hao W Sun R Li L Jiang C. A simple nomogram for assessing the risk of IgA vasculitis nephritis in IgA vasculitis Asian pediatric patients. Sci Rep. (2022) 12(1):16809. 10.1038/s41598-022-20369-3 36207379 PMC9547060

[30] He X Yin W Ding Y Cui S-j Luan J Zhao P. Higher Serum angiotensinogen is an indicator of IgA vasculitis with nephritis revealed by comparative proteomes analysis. PLoS One. (2015) 10(6):e0130536. 10.1371/journal.pone.0130536 26098644 PMC4476708

